# Body-Related Attitudes, Personality, and Identity in Female Adolescents with Anorexia Nervosa or Other Mental Disorders

**DOI:** 10.3390/ijerph19074316

**Published:** 2022-04-04

**Authors:** Melanie Achermann, Juliane Günther, Kirstin Goth, Klaus Schmeck, Simone Munsch, Lars Wöckel

**Affiliations:** 1 Center of Child and Adolescent Psychiatry and Psychotherapy, Clienia Littenheid AG, 9573 Littenheid, Switzerland; juliane.guenther@clienia.ch; 2 Department of Psychology, University of Fribourg, 1701 Fribourg, Switzerland; simone.munsch@unifr.ch; 3 Child and Adolescent Psychiatry, Research Department, University Hospital of Psychiatry Basel, University of Basel, 4002 Basel, Switzerland; kirstin.goth@upk.ch (K.G.); klaus.schmeck@unibas.ch (K.S.)

**Keywords:** body dissatisfaction, drive for thinness, adolescence, personality, identity, anorexia nervosa, bulimia nervosa, depression, borderline personality disorder, ADHD

## Abstract

The psychological integration of body-related attitudes (BodyRA) is a critical developmental task in adolescence. Adolescents must adapt to their changing body image and body satisfaction. For young people, BodyRA (body dissatisfaction, bulimia, and drive for thinness) are connected to insecurities, which can disturb identity integration and personality development. Our goal was to evaluate the importance of BodyRA also for other mental disorders other than anorexia nervosa (AN), and the association between BodyRA with temperament and personality traits and identity diffusion. Data for the period of 2012 to 2019 were retrospectively analyzed from a convenience sample of patients in a child and adolescent psychiatric hospital (*n* = 114). The patients were 13 to 17 years of age and had a BMI of 11.9–36.1 kg/m^2^. As expected, BodyRA were found to be more pronounced in AN, as well as in borderline personality disorder (BPD), depression (DD), and attention deficit hyperactivity disorder (ADHD). BodyRA correlated significantly with internalizing problems in patients with DD (*r* = 0.428–0.565, *p* < 0.01) and BPD (*r* = 0.680, *p* < 0.01) as well as with BMI (*r* = 0.404, *p* < 0.01) in patients with DD. Moreover, we detected significant correlations with impaired identity development in patients with DD (*r* = 0.482–0.565, *p* < 0.01) and BPD (*r* = 0.681–0.703, *p* < 0.01). BodyRA also correlated significantly with the personality traits of harm avoidance (*r* = 0.377–0.541, *p* < 0.01) and self-directedness (*r* = −0.537–−0.635, *p* < 0.01) in DD. These personality traits and bulimia were used as predictors for identity diffusion in the investigated disorders of this study. We conclude that BodyRA, harm avoidance and self-directedness are associated with identity development in adolescent females with mental disorders.

## 1. Introduction

Adolescence is both mentally and physically a challenging developmental phase [1,2,3] and can lead to body-related dissatisfaction and negative body image, which are often associated with feelings of insufficiency and decreased social acceptance [4,5,6,7,8]. O’Dea and Abraham [9] found that puberty—a developmental period characterized by the acceleration of height growth, the appearance of secondary sexual characteristics, body composition alterations, and increasing circulating hormone levels—is a risk factor for body dissatisfaction and psychological problems in girls. It is assumed that a large percentage of girls (24–90%) are affected by body dissatisfaction [10]. In this context, increased body weight is associated with poor body image [11,12,13,14]. In ICD-10 and ICD-11, the primary features of anorexia nervosa (AN) and bulimia nervosa (BN) are a distorted body image and high dissatisfaction with one’s body shape and weight [15,16]. Besides focusing on body image disturbances in AN, DSM-5 emphasizes self-evaluation, which can be influenced by body shape and weight in both AN and BN [17]. It was shown that post-menarche girls have a significantly higher Body Mass Index (BMI) than pre-menarche girls of the same age [18]. This factor increases the probability of engaging in dieting and increased exercise [19,20,21,22,23,24]. Frost and McKelvie [25] found that physical acceptance and body satisfaction are predictors of self-esteem and detected evidence that self-esteem in female students is related to weight satisfaction. Rosenblum and Lewis [26] observed a low but significant correlation between BMI and body dissatisfaction in adolescents between 13 and 15 years of age but no relation between those aged between 15 and 18 years. Compared to late onset AN, AN with an early onset seems to be associated with a more disturbed personality, higher body dissatisfaction, and a drive for thinness with an ascetic lifestyle [27]. Stice et al. [28] introduced a dual-pathway model to help explain the development of eating disorders (ED) by investigating the interrelationships of sociocultural influences, diet, and negative affect. For BN, Stice [29] demonstrated that elevated pressure to be thin and internal thin-ideal representation foster body dissatisfaction, which also promotes dieting and negative affect, consequently increasing the risk for bulimic pathology. Subsequently, Stice et al. [30] enhanced the dual-pathway model for potent predictors of AN, BN, binge eating disorder (BED), and purging disorder as well as the amplifiers of these relations. Furthermore, the authors were able to support the temporal succession of risk factors in the dual-pathway model of ED [31]. Body dissatisfaction not only seems to be a predictor for disordered eating behaviors and the development of ED [32] but also happens to be important in the course of other mental disorders [10]. Stice and Bearman [33] suggested that the impact of being thin, internal thin-ideal representation, body dissatisfaction, and dietary and bulimic behavior predicted increased depressive symptoms. 

Some studies instead suggested a strong relationship between body image and depressive disorders (DD), both for young people with obesity e.g., [34] and for non-overweight adolescents [35]. A negative evaluation of one’s own physical appearance, concern for obesity, and body dissatisfaction are also found in other mental disorders [36]. Christian et al. [37] observed a low but significant correlation between the drive for thinness and bulimic symptoms in female and male undergraduate students compared to the core symptoms of attention deficit hyperactivity disorder (ADHD). A Swedish nationwide population study revealed a positive genetic correlation of ADHD polygenic risk scores with the Eating Disorder Inventory (EDI) subscales of drive for thinness and body dissatisfaction [38]. Literature concerning externalizing disorders and body-related attitudes (BodyRA) is very rare and conflicting. Baker et al. [39] reported no significant associations between the Youth Self Report (YSR) scale for externalizing behavior and drive for thinness and body dissatisfaction. On the other hand, Marmorstein et al. [40] found that previous externalizing behavior could predict increased levels of weight concern, body dissatisfaction, and the use of unsuitable compensatory behaviors. Moreover, adolescents with symptoms of borderline personality disorder (BPD) often exhibit body dissatisfaction. Accordingly, patients with BPD also have a high prevalence of ED, with 29% for AN [41]. 

AN and BN have both a similar comorbidity profile in personality disorders. More than one half of patients with AN and BN have comorbid personality disorders, with a high prevalence of BPD (19 vs. 25%) [42]. Thus, BPD was described as a factor influencing future weight concerns [43]. Numerous studies with adults revealed a correlation between temperament, character, or personality traits with personality disorders or ED [41,44,45]. Therefore, as expected, the temperament dimensions in adolescents with an ED were similar to those in adults with an ED [46]. 

Furthermore, impulsivity appears to be a relevant factor that informs the temperament of bulimic patients [47] while anorexic symptoms are associated with persistent temperamental characteristics [47]. BN patients scored higher for novelty seeking but lower for persistence than patients with AN_R_ (restrictive type) [48]. In contrast to patients with AN_R_, both patients with BN or AN_P_ (purging type) achieved lower values for self-directedness [48]. Further, the impairment of identity development is different in adolescent patients with different forms of mental disorders [49]. Patients with personality disorders showed high identity diffusion compared to a healthy sample [49]. Identity integration is regarded as a central development task in which both personal needs and social expectations must be met [50] within the framework of adolescent development, which is regarded as a precursor of the ability to form satisfactory, intimate bonds through the consolidation of identity in adolescence [51]. Adolescents must develop a realistic self-image (e.g., body image), which can be complicated by unstable self-esteem [32]. Identification with peers in an unrealistic way can lead to a distortion of ED-related attributes [51]. Mas et al. [52] showed that self-esteem and perfectionism have a mediating role in the relationship between personality traits and body dissatisfaction, dietary restraint and purging behavior in female adults. According to the authors, body image is a predictive factor for self-esteem and self-esteem showed a main mediating effect between personality traits and ED, and perfectionism is a mediator regarding the effect of borderline personality traits on ED and self-esteem [52]. Verschueren et al. [53] showed that patients with an ED had difficulties with identity-related topics. Patients with an ED also showed increased identity diffusion compared to people without this diagnosis [43,52,54]. It is assumed that body weight is controllable and culturally recognized, which is why these adolescents use their bodies for self-definition, wherein the urge to change one’s own body can actually represent a search for individuality [55]. Since drive for thinness, body dissatisfaction, and/or bulimic behavior is often observed in BPD, DD, and ADHD, we suspect that BodyRA play an important role not only in AN but also in other mental disorders, as the prevalence levels suggest. Another aim of this study was to clarify the question of whether there is an association between BodyRA, identity, or personality in AN or other mental disorders in female adolescents.

## 2. Materials and Methods

### 2.1. Participants

Data were retrospectively analyzed using a sample of inpatients and outpatients of a child and adolescent psychiatric hospital from the period of 2012–2019 (*n* = 114; Clienia Littenheid AG, Littenheid, Thurgau, North-East Switzerland). The patient sample consisted of female adolescents aged 13 to 17 years (15.6 ± 1.1 years), with a BMI of 11.9–36.1 kg/m^2^ (21.6 ± 4.9 kg/m^2^) and a diagnosed mental disorder. Only female adolescents were included because the prevalence and risk factors for disordered symptoms are very different between males and females [56,57]. 

In total, 28 patients were diagnosed with an ED (15.4 ± 1.1 years, BMI = 18.1 ± 4.1 kg/m^2^), and 86 patients were treated due to a mental disorder without an ED (15.7 ± 1.1 years, BMI = 22.7 ± 4.6 kg/m^2^). All patients were surveyed to identify development (Assessment of Identity Development in Adolescence (AIDA)), ED characteristics (Eating Disorder Inventory 2 (EDI-2)), syndrome scales (Youth Self Report (YSR)), and/or personality characteristics (Junior Temperament and Character Inventory (JTCI)) as part of their diagnostic procedure. These factors were used to assign each patient to one of the diagnostic groups based on the characteristic main diagnosis (AN_R_ (ICD-10 F50.00, *n* = 16), DD (ICD-10 F32, *n* = 46), emotionally unstable personality disorder (BPD) (ICD-10 F60.3, *n* = 14), including impulsive type (ICD-10 F60.30, *n* = 2) and borderline type (ICD-10 F60.31, *n* = 12), ADHD (ICD-10 F90.0, *n* = 14), and conduct disorder (CD) ((with (ICD-10 F90.1, *n* = 6) and without ADHD (ICD-10 F91, *n* = 4)).

Most inpatients with ADHD showed greater severity in clinical expression and depressive behaviors. The included ADHD patients had *t*-values > 63 (clinically conspicuous) on a superordinate scale for internalizing behavior problems (YSR) (mean *t*-value of internalizing problems = 73.7 ± 6.7). It addresses a subgroup of ADHD patients. The number of patients diagnosed with AN purging type (AN_P_, ICD-10 F50.01), atypical AN (AN_A_, ICD-10 F50.1), BN (ICD-10 F50.2), and other ED (ICD-10 F50.8, corresponding to BED in DSM 5) were each smaller (*n* < 5) and not included within this study. The characterization of the groups used in this study is shown in Table 1. Psychological assessments and questionnaires were applied to all patients during the treatment and admission phase and were not conducted before the 5th week after beginning treatment. In addition, a diagnostic procedure was performed in patients with an ED when the process of weight gain was successful and eating behavior began to normalize. The weight was measured and the BMI was calculated for this study at the time of psychological testing. The diagnostic assignment was made by expert opinions based on the ICD-10 diagnostic criteria of psychiatric disorders. A further-disorder specific diagnosis was performed individually, including structured interviews, tests of attentional performance, intelligence tests, and questionnaires. Weekly consensus conferences were held to establish clinical ICD-10 discharge diagnoses according to the multiaxial classification of child and adolescent mental disorders [13]. This diagnostic test battery was not applied to patients with psychosis, developmental disabilities, acute suicidality, or endangerment. 

### 2.2. Questionnaires

#### 2.2.1. Eating Disorder Inventory 2 (EDI-2)

The EDI-2 is a multidimensional self-report questionnaire used to assess ED psychopathology in adolescents (>12 years) and adults, such as AN (restricting and binge-eating/purging type), BN, and ED not otherwise specified (including BED). Garner published the first version of the EDI in 1983 [58]. In 1991, Garner published a revised version (EDI-2) that retained the original 64 items with an additional 27 items describing three new subscales to provide the subscale scores and total score. The body-related subscales “drive for thinness”, “bulimia”, “body dissatisfaction”, and total score were evaluated in this study. The EDI-2 is considered to be an objective, reliable, and valid questionnaire. The Cronbach’s alpha was between 0.73 and 0.93 for anorectic and bulimic patients [59].

#### 2.2.2. Junior Temperament and Character Inventory (JTCI)

The JTCI is a reliable and valid method to capture the basic personalities of children and adolescents according to Cloninger’s revised biopsychosocial personality model [60]. Generally, this assessment can be used for both clinical and nonclinical samples. The German JTCI test family is available in various age versions [61]. The JTCI 3–6R and the JTCI 7–11R are parent forms, and the 12–18R is a self-report form for adolescents. The JTCI 12–18R used in this study consists of 103 items and assesses four temperament scales (novelty seeking, harm avoidance, reward dependence, and persistence), as well as three character scales (self-directedness, cooperativeness, and self-transcendence). The Cronbach’s alpha was between 0.79 and 0.85.

#### 2.2.3. Assessment of Identity Development in Adolescence (AIDA)

The AIDA (Assessment of Identity Development in Adolescence) is a questionnaire that uses 58 five-point-scale items to assess identity development in terms of impairments in personality functioning among adolescents aged 12 to 18 via self-reporting [62]. This test enables dimensional differentiation between healthy and impaired identity development, which is assumed to be associated with a high risk of a current personality disorder, especially BPD, in line with the new definition of personality disorders in the AMPD (Alternative Model of Personality Disorders) (DSM-5 [17]) and the upcoming ICD-11 [16]. 

The construction of the AIDA model focused on clinical validity. All items were added together to obtain the total scale of identity diffusion. For descriptive reasons, the total scale was divided into the two domains of discontinuity and incoherence (primary scales), each containing three different aspects of identity development (subscales). This format reflects the theoretical origins and complexity of the concept and is intended to facilitate a differentiated interpretation of the results and specific therapy planning. For screening, it is sufficient to consider the total score. The scale reliabilities were well with Cronbach´s alpha (0.94 (total), 0.87 and 0.92 (primary), and 0.69 to 0.84 (subscale level)). The AIDA total score (identity diffusion) differed at a high significance level with a relevant effect size (d = 2.6 standard deviations) between the general population (*n* = 1437) and a subsample (*n* = 25) of patients diagnosed with BPD (SCID-2). The difference for patients with other personality disorders achieved a good effect size (d = 2.0) compared to that for patients without a personality disorder (d = 0.9; patients with internalizing or externalizing disorders). This result indicates the ability of the construct assessed by the AIDA to describe impairments associated with BPD, as well as other personality disorder pathologies [62,63,64].

#### 2.2.4. Youth Self Report (YSR)

The YSR assesses competencies and behavioral and emotional problems in individuals aged 11 to 18 years [65]. The YSR is a self-rating form parallel to the Child Behavior Checklist (CBCL) and serves as a screening instrument to assess the psychopathology of children and adolescents in parent reports. The YSR was translated into German, and psychometric properties were successfully tested [66]. The YSR is structured using eight problem scales based on 118 items (anxious/depressed, withdrawn/depressed, somatic complaints, social problems, thought problems, attention problems, rule-breaking behavior, and aggressive behavior), which are summed using up to three superordinate scales (internalizing behavior problems, externalizing behavior problems, and total behavior problems). The Cronbach’s alpha was set to >0.80 for internalizing and externalizing behavior problems.

### 2.3. Statistical Analyses

One-way analysis of variance (ANOVA) was conducted with Bonferroni post hoc tests analysis to examine the differences between groups. Based on the skewness and kurtosis statistics, the variables were judged to be normally distributed. The Levene test was used for examining the equality of variance (homogeneity). The alpha level was set at ≤0.01 (two-tailed) for all statistical comparisons. The Pearson´s correlation coefficients between BodyRA, BMI, YSR syndrome scales, the total scale of identity diffusion, and personality characteristics were computed (two-tailed). To determine if the BodyRA, temperament, and character scales could explain a statistically significant amount of variance in identity diffusion (dependent variable), we performed stepwise regression analyses. These analyses were calculated separately for each patient group. Statistical calculations were performed using SPSS Version 24 (IBM Corp. in Armonk, NY, USA).

## 3. Results

### 3.1. Group Differences in BodyRA (EDI-2)

Variance analyses were significant (α ≤ 0.01) only on the total EDI-2 score (df = 4, F = 3.98, *p* = 0.005). AN_R_ and BPD showed the highest *t*-values on the scale of drive for thinness. The bulimia scale increased (*t*-value > 60) in BPD, DD, and ADHD and was the highest for BPD without significant differences between these disorder groups. The body dissatisfaction scale only increased in BPD. The BPD group was the only group that showed elevated values on all BodyRA scales. Nevertheless, the *t*-values did not differ between any groups in BodyRA on α-level ≤ 0.01. Patients with CD showed no relevant differences compared to the sample of a nonclinical population of female college students in EDI-2 in terms of BodyRA and the total EDI-2 score. The *t*-values for total EDI-2 score were above 60 for AN_R_, DD, BPD and ADHD, and this score was significantly increased in BPD compared to CD, likely due to integration of the scales into EDI-2 (e.g., ineffectiveness, interpersonal distrust, impulse regulation, and social insecurity), which were often increased in this patient group. The BPD group, moreover, showed the highest *t*-value for total EDI-2 in this study (Table 2).

### 3.2. Group Differences in Personality Traits (JTCI)

One-way analysis of variance yielded significant differences between the mental-disorder groups in two temperament scales (novelty seeking and persistence). AN_R_ had the lowest *t*-values in novelty seeking, which were significantly lower than those for CD. Furthermore, AN_R_ presented the highest *t*-values in persistence and cooperativeness and significant differences within persistence groups. Harm avoidance and self-directedness revealed a reverse phenomenon. *t*-values in harm avoidance and self-directedness were the most prominent in BPD. In the ANOVA, no significant differences were observed in the scales of reward dependence, cooperativeness, and transcendence (Table 3).

### 3.3. Group Differences in Identity Development (AIDA)

The AIDA performance showed significant differences in one-way analysis of variance (ANOVA) in the total scale of identity diffusion; the primary scales of discontinuity and incoherence; and the subscales of perspectives and attributes, emotional self-experience, self-concepts, and cognitive self-experience (Table 4). The highest and most conspicuous *t*-values on all scales were observed in BPD and were increased compared to the sample with healthy students [49]. AN_R_ and CD showed the lowest *t*-values on all these scales and were not significantly different from one another. Compared to BPD, we found significantly lower *t*-values in CD on most subscales. Moreover, compared with DD, BPD, and ADHD, we observed significantly lower *t*-values in CD for the subscale of emotional self-experience.

### 3.4. Covariations between BodyRA, Personality, and Identity

We observed some significant correlation coefficients at a 0.01 level between BodyRA and temperament and character scales (Table 5) according to JTCI in the DD group. This result indicates negative correlations between the drive for thinness, bulimia, and body dissatisfaction and self-directedness, as well as between body dissatisfaction and persistence. A positive correlation was found between BodyRA and harm avoidance. Significant correlation coefficients between BodyRA and identity diffusion and primary scales of identity were also detected in DD (in Table 5, only the total scale identity diffusion is shown). Drive for thinness and bulimia were correlated with the identity scales in BPD. Furthermore, we observed significant correlations between internalizing problems (YSR) and all BodyRA in DD, as well as between internalizing problems and drive for thinness in BPD. Significant correlations between BodyRA (body dissatisfaction) and BMI were only found in DD. Otherwise, there were no significant correlations between BMI and any of the investigated parameters (not shown). 

Analysis of hierarchical regression was performed in the groups of this study separately (dependent variable = identity diffusion) (Table 6). At a 0.01 level, one model was calculated in each group (AN_R_, DD, BPD, ADHD, and CD). With 69% variance, self-directedness explained the identity diffusion in AN_R_. In DD and ADHD, identity diffusion was also explained by self-directedness, with a variance of 57% and 74%. In BPD, bulimia explained identity diffusion with a variance of 45% and harm avoidance with 75% in CD. The R^2^ change was found to be significant in all models (sig. F change between 0.001 and 0.005). Excluded variables were BodyRA (except bulimia in BPD), temperament, and character scales (except for self-directedness and harm avoidance in the aforementioned groups) and BMI.

## 4. Discussion

The results of our study show that BodyRA are common among female adolescent patients with AN and among patients with BPD, DD, and ADHD. Drive for thinness, which is defined as the desire to be thinner or to be objectively thin [67], and the tendency to engage in bulimic behavior is, therefore, particularly noteworthy. As would be expected in accordance with the literature, we found higher scores on the drive for thinness subscale in AN. Adolescents and young adults with AN were characterized by a significantly increased drive for thinness, especially when co-morbid with more severe anxiety and depression [68]. Anxious and depressive symptoms are frequent among patients with an ED [68]. Solmi et al. [69] concluded that depressive and anxious symptoms, interpersonal functioning, and ineffectiveness play a central role in maintaining symptomatology in ED. As a major feature of ED, the drive for thinness is one of several risk factors in the development of ED [67] and has a prognostic role in determining the outcome of an ED [70]. Prost-Lehmann et al. [71] demonstrated that body dissatisfaction is correlated with ED outcomes, but its impact is mediated by the drive for thinness. Rozenblat et al. [72] detected a significant association between depressive symptoms and lower emotional control and drive for thinness, as well as bulimic behavior. The authors emphasized that DD and emotional control could play key roles in the adolescent drive for thinness and bulimic behavior. Our results are consistent with the abovementioned findings, especially those reported by Penas-Lledo et al. [68]. 

We observed significant correlations between BodyRA, particularly with regard to the drive for thinness and internalizing problems on YSR scores. Interestingly, this phenomenon mostly affected DD and BPD. It cannot be ruled out that significant effects in DD and BPD are emphasized by the very small sample sizes of most groups (see limitations). Our study yielded significantly high values on the drive for thinness subscale in BPD and marginal values in ADHD, which are both recognized to feature emotional dysregulation as a core feature. Fiore et al. [73] demonstrated that drive for thinness is significantly associated with anxiety control over emotional reactions and external threats, as well as with emotional dysregulation in anorectic patients. The authors reasoned that anxiety control mediates the association between emotional dysregulation and drive for thinness. This mediational model could be similar for BPD and ADHD. Higher scores on the bulimia subscale were observed for BPD, DD, and ADHD. Although the bulimia subscale of EDI-2 records cognition and behaviors related to BN, the full diagnostic criteria of BN were not requested in the questionnaire. Nevertheless, the results of our study correlate well with the literature. A co-morbid diagnosis of BN is often reported in BPD. Zanarini et al. [74] observed co-morbid BN in 25.6% of in-patients with BPD. Research regarding comorbidities shows that BN diagnoses are often found in individuals with depressive symptoms [75]. Therefore, the lifetime prevalence of an ED among female inpatients and outpatients with BN and depression is expected to range between 9.4% and 21.2% [75]. ADHD girls were 3.6 times more likely to meet the criteria for any ED [76] and 5.6 times more likely to develop BN compared to the controls [77]. 

Significantly greater rates of BN were identified in women with ADHD (12%) than in women without ADHD (3%) [77]. According to a Swedish nationwide population study, the prevalence of BN in female individuals with ADHD was 1.8% compared to 0.4% for females without ADHD. The odds ratios (OR) for female individuals with ADHD revealed an increased risk for EDs not specified (OR = 4.63) and for BN (OR = 4.94) [37]. Girls with ADHD and depressive symptoms, anxiety, and disruptive disorders are at higher risk of developing BN [78]. Our findings revealed an association between internalizing behavior problems and BodyRA in DD and partly in BPD. These results are in close agreement with those obtained by Stice and Whitenton [79] and Polivy and Herman [80], who reported an association between body dissatisfaction, anxiety, DD, and ED. Fernández-Bustos et al. [81] noted that gender and BMI are crucial variables when determining body dissatisfaction in adolescents. Among females, body dissatisfaction commonly reaches high levels during adolescence [82]. Overweight and underweight are both risk factors for body dissatisfaction [75]. Indeed, Kostanski et al. [83] observed a linear association between BMI and dissatisfaction in females. Our data are in accordance with those of previous studies and confirm an association between BMI and body dissatisfaction in DD. However, in AN_R_, this association failed to appear. This result may be due to cognitive beliefs and distortions in AN. The results of this study are mostly consistent with those of previous studies that investigated mental disorders and temperament using character scales. Harm avoidance increased and self-directedness decreased in most mental disorders in this study, except for CD. Likewise, this trial revealed decreased self-directedness in AN_R_. Some authors have indicated that increased harm avoidance can be observed in BPD [45,84,85], BPD combined with ADHD [86], DD [87], AN_R_ [46], and children with withdrawn behavior [88]. Furthermore, data from the literature described strong correlations between depressive symptoms and high scores for harm avoidance. Reduced self-directedness was reported in BPD [45,84,85], ADHD [89,90,91], ADHD combined with oppositional defiant disorder (ODD) [92], DD [87], and AN_R_ [43,44,46,93]. Fassino et al. [44] underlined that low self-directedness is a risk factor for susceptibility to social pressures for slenderness and highlighted the prognostic value of this factor for the long-term outcome of AN. In contrast, our data do not confirm previous studies demonstrating high levels of novelty seeking in BPD [45,86], ADHD [90,91], BN [44,93], and ODD [94]. The results of our study indicated novelty-seeking levels within a normal range in nearly all patient groups. Only patients with AN_R_ showed decreased levels of novelty seeking, which was likewise described in studies from Hueg et al. [48] and Fassino et al. [44]. Fassino et al. [44] supported the hypothesis that a combination of low novelty seeking, high harm avoidance, and high persistence is a characteristic trait of AN_R_. Zohar et al. [95] found that stability of temperament and character traits from ages 12 to 16 years were only found in half of the studies on adults over their life spans. The authors also found that novelty seeking increased from age 12 to 14 before declining after age 14. In addition, the authors observed that girls exhibited significantly lower levels of novelty seeking and higher levels of harm avoidance, reward dependence, and persistence compared to boys. The sample of this study included only girls with a mean age of 16.0 ± 1.3 years, which might be an influencing factor explaining the inconspicuous novelty seeking levels. Furthermore, our sample of female BPD patients consisted mainly of the borderline type and not the impulsive type, which might also explain the lower levels of novelty seeking.

Goth et al. [49] evaluated the psychometric properties of the AIDA questionnaire and assessed the convergent and discriminant validity between AIDA and JTCI 12–18R. The authors showed high negative correlations between all identity scales and self-directedness (−0.59 to −0.76), as well as medium to high positive correlations between the primary identity scales (discontinuity and incoherence) and harm avoidance (0.33 to 0.60). The other temperament and character scales revealed low or very low correlations with AIDA scales, with novelty seeking and cooperativeness exhibiting the lowest correlations. In line with Jung et al. [96], the *t*-values of identity diffusion (*t* = 72.0) and primary scales (discontinuity *t* = 74.3, incoherence *t* = 68.4) of the BPD groups in our data were consistent with the *t*-values (mean *t* = 73) in the respective personality disorder groups, mostly BPD. Both groups had the highest scores on all AIDA scales compared to other mental disorders. Patients with DD also presented elevated scores; however, these scores remained marginally lower than those in the BPD group. This outcome is in contrast with the results of Jung et al. [96] who noted that the personality disorder group discriminated significantly compared to the internal disorder group. These different results may be due to the heterogeneous composition of the internal disorder group, which included DD, anxiety disorders, and emotional disorders. Furthermore, our findings revealed differences in identity diffusion between ADHD (*t*-values > 60) and CD (*t*-values within a normal range), unlike the study of Jung et al. [96], which merged ADHD and CD into an external disorder group, which could explain the discrepancy in the *t*-values between the two studies. Furthermore, the ADHD group in our study was characterized by increased *t*-values for internalized behavior problems. No pathological scales for patients with external disorders and CD on the identity and temperament and character scales can be probabilistically attributed to consistent self-image and autonomous self-awareness [96]. Likewise, the *t*-values for patients with AN_R_ were within a normal range on the identity scales, except for consolidating in relationships, roles, perspectives, attributes and discontinuity. The self-assessment of patients with AN_R_ revealed a consistent self-image, autonomy, and cognitive self-experience. This result is surprising since self-competence and self-appreciation are frequently low among patients with AN. A possible explanation for this result could be the interaction between a distorted body image self-schema function and the impaired emotional recognition often found in AN [97], combined with cognitions typically found in AN, e.g., over-evaluating one’s achievements. Fassino et al. [44] suggested that a combination of a low level of self-directedness and a high level of harm avoidance reduces one’s coping skills against stressors and negative life events. In our study, the ratio between low levels of self-directedness and high levels of harm avoidance was even more pronounced in mental disorders, such as ADHD, DD, and BPD but not as distinct in AN_R_ and CD. Nevertheless, the hierarchical regression analysis revealed models indicating that these character and temperament scales and bulimia can explain between 45 and 75% of variance as predictors for identity diffusion in AN_R_, DD, BPD, ADHD, and CD. In addition, drive for thinness was significantly correlated with identifying diffusion in DD and BPD. Prost-Lehmann et al. [72] found that drive for thinness has a high impact on mediating body dissatisfaction, which is associated with self-esteem and, in a broader sense, enables or prevents the development of a positive self-concept. Our results are also in accordance with the dual-pathway model of Stice [31] and suggest that the pressure to be thin and thin-ideal internalization occurs first with disorder-predictive levels, followed by body dissatisfaction, dieting, and/or negative effect, ending with the onset of an ED. Together with the temperament and character scales, this model could provide an explanation for the high risk of developing an eating pathology and for the high rates of comorbidities between BPD, DD, and ADHD and an ED. 

Interestingly, hierarchical regression in AN_R_ provided a model that could explain 69% of the variance with self-directedness as a predictor for identity diffusion. However, BodyRA were not included in this model. Possibly, the development of identity in patients with AN_R_ is different from that in patients without AN. This hypothesis suggests that we found no correlations between temperament and character scales, drive for thinness, and bulimia/body dissatisfaction in AN_R_, except for self-directedness. On the other hand, BodyRA correlated with self-directedness and harm avoidance in patients with DD and identity diffusion in DD and partly in BPD. These significant correlations are of high interest since there is some evidence that drives for thinness [98], bulimia [99], self-directedness, and harm avoidance are associated with serotonin, specifically 5-HT_2_ receptor sensitivity [100] or serotonin transporter polymorphism (5-HTTLPR) [101]. It is widely accepted that serotonin is involved in the pathophysiology of ED as well as in DD, BPD, and ADHD. We suggest that BodyRA together with a specific temperament and character traits can serve as predictors for the development of identity in female adolescents with mental disorders. Serotonin may possibly influence this interaction. Further research is necessary to investigate this question.

### Limitations

The design of this study was retrospective. The sample size of each group in terms of mental disorders and AN_R_ differed and was generally small. Because of the very small sample sizes for groups of patients with an ED other than AN_R,_ these groups were not included in further calculations. Possible cohort effects related to differences between the patient groups. The composition of the CD group, moreover, was not homogeneous (with and without ADHD), and statistical analyses were performed using *t*-values. Because of the very small sample sizes for most disorder groups (except DD) the calculations, therefore, have a very low power to detect significant correlations. For this reason, significant correlations, mainly in the DD group, could be due to the larger sample size. We observed some correlation coefficients (r > 0.5), which were not significant due to small sample sizes, but they would not change our main results and conclusions crucially.

## 5. Conclusions

This study examines, for the first time, the association between personality traits and attitudes specific to ED (body-related attitudes = BodyRA) and the development of identity pathology (AIDA) in female adolescents with AN_R_ and other mental disorders. We showed that BodyRA (drive for thinness, bulimia, and body dissatisfaction) is increased (*t*-values > 60) not only in AN_R_ but also in BPD, DD, and ADHD. Increased values of BodyRA might be a feature of adolescence since the development of body image in girls changes during and after puberty, in which BMI plays a role. Accordingly, we found a moderate and significant correlation between BMI and body dissatisfaction in female adolescents with DD. Another explanation for the increased values of BodyRA could be an overlap between psychopathology and characteristics in personality between ED, BPD, DD, and ADHD. These disorders are often comorbid with each other and may explain the high comorbidity rate with eating disorders. Hence, patients in our study with DD and BPD noted significant correlations between internalizing problems and BodyRA. Personality, when assessed with the JTCI, revealed increased harm avoidance and decreased self-directedness in BPD, DD, and ADHD. BodyRA correlated with harm avoidance and self-directedness in DD as well as with self-directedness in AN_R_. Moreover, we observed significant correlations between BodyRA and identity diffusion in DD and BPD but not in AN_R_. Both CD and AN_R_ generally presented inconspicuous values on the identity scales, which might be explainable using disorder-specific characteristics. Self-directedness is a predictor of identity diffusion in AN_R_, DD and ADHD, harm avoidance in CD, and bulimia in BPD. The results of this study support the hypothesis that BodyRA play a key role in the onset of ED pathology in mental disorders. We conclude that for specific BodyRA, respectively, personality characteristics have a high impact on the development of identity in female adolescents with mental disorders investigated in this study. However, the results are based on very small sample sizes and further studies are needed.

## Figures and Tables

**Table 1:** Characterization of patient groups: *Anorexia nervosa* restrictive type (AN_R_), depressive disorder (DD), borderline personality disorder (BPD), attention deficit hyperactivity disorder (ADHD), conduct disorder (CD), body mass index (BMI).

.	AN_R_	DD	BPD	ADHD	CD
ICD-10 (F)	50.00	32	60.3	90.0	90.1/91.0
n	16	46	14	14	10
	**Means and standard deviations**				
Age (years)	15.6	15.7	16.1	15.6	15.3
	±1.1	±1.1	±1.0	±1.3	±1.1
BMI (kg/m^2^)	16.1	22.1	23.5	23.0	24.1
	±1.8	±3.9	±4.4	±5.3	±7.0

**Table 2:** Means and standard deviations of *t*-values for Eating Disorder Inventory 2 (EDI-2). Body-related subscales (drive for thinness, bulimia, and body dissatisfaction) and the total score were selected. One-way analysis of variance (ANOVA) was performed over EDI-2 subscales and the total score for different mental-disorder groups. Bonferroni post hoc tests were calculated between groups. Only one sample with Bonferroni post hoc test calculations features significant values between groups on α-level ≤ 0.01 (bold print); α-level ≤ 0.05 (light print); n.s. = not significant.

EDI-2 Scales	Means and Standard Deviations					F	AN_R_	DD	BPD	ADHD
	AN_R_	DD	BPD	ADHD	CD	*p*	CD	CD	CD	CD
Drive for thinness	65.1	57.9	66.4	59.7	49.8	3.25	0.044	n.s.	0.028	n.s.
	±9.9	±13.9	±12.9	±14.5	±10.3	0.015				
Bulimia	56.7	61.2	64.4	61.6	55.5	1.46	n.s.	n.s.	n.s.	n.s.
	±12.5	±10.3	±10.5	±12.3	±11.9	n.s.				
Body dissatisfaction	59.7	56.7	66.4	56.9	51.6	2.88	n.s.	n.s.	0.026	n.s.
	±10.7	±12.1	±9.3	±12.6	±11.1	0.027				
Total score	68.8	67.0	70.3	69.1	55.1	3.98	0.015	0.013	**0.006**	0.013
	±8.3	±11.4	±8.3	±8.3	±11.9	**0.00** **5**				

**Table 3:** Means and standard deviations of t-values of scales of Junior Temperament and Character Inventory (JTCI) for mental-disorder groups. One-way analysis of variance (ANOVA) and Bonferroni post hoc tests analysis. Only samples with Bonferroni post hoc tests calculations that feature significant values are included (α-level ≤ 0.01 (bold print), α-level ≤ 0.05 (light print)), n.s. = not significant.

JTCI Scales	Means and Standard Deviations					F	AN_R_	AN_R_	AN_R_	DD	BPD
	AN_R_	DD	BPD	ADHD	CD	*p*	DD	ADHD	CD	CD	CD
Temperament scales											
Novelty seeking	39.7	45.5	49.2	50.4	53.6	5.19	n.s.	0.012	**0.002**	n.s.	n.s.
	±9.4	±9.1	±9.6	±7.5	±6.7	**0.001**					
Harm avoidance	59.3	62.9	64.6	61.1	50.5	3.47	n.s.	n.s.	n.s.	**0.009**	0.015
	±9.8	±9.2	±11.6	±13.0	±11.0	0.011					
Reward dependence	44.1	46.3	46.1	45.2	49.6	0.36	n.s.	n.s.	n.s.	n.s.	n.s.
	±13.3	±11.8	±10.5	±11.5	±9.9	n.s.					
Persistence	53.6	42.7	46.3	40.9	49.1	4.55	**0.003**	**0.008**	n.s.	n.s.	n.s.
	±12.1	±7.6	±11.5	±12.4	±11.4	**0.002**					
Character scales											
Self-directedness	39.4	36.5	32.9	34.4	47.7	3.06	n.s.	n.s.	n.s.	n.s.	0.021
	±13.5	±10.4	±9.2	±13.0	±12.0	0.020					
Cooperativeness	55.1	53.2	54.5	52.7	51.3	0.28	n.s.	n.s.	n.s.	n.s.	n.s.
	±9.3	±9.7	±10.6	±11.2	±9.4	n.s.					
Self-transcendence	45.9	51.4	50.9	51.8	50.5	1.02	n.s.	n.s.	n.s.	n.s.	n.s.
	±7.3	±10.7	±9.6	±9.5	±9.5	n.s.					

**Table 4:** Means and standard deviations of t-values for the total scale of identity diffusion, primary scale of discontinuity and incoherence, and subscales of Assessment of Identity Development in Adolescence (AIDA) for mental-disorder groups. One-way analysis of variance (ANOVA) was conducted with Bonferroni post hoc test analysis to examine the differences between groups. Only samples with Bonferroni post hoc test calculations that included significant values are listed (α-level ≤ 0.01 (bold print), α-level ≤ 0.05 (light print)); n.s. = not significant.

AIDA Scales	Means and Standard Deviations					F	AN_R_	DD	BPD	ADHD
	AN_R_	DD	BPD	ADHD	CD	*p*	BPD	CD	CD	CD
Identity Diffusion	59.7	66.4	72.0	68.6	54.8	5.07	0.028	0.031	**0.00** **3**	0.032
	±11.7	±10.4	±8.3	±11.2	±14.8	**0.00** **1**				
Domain 1 - Discontinuity	61.4	68.6	74.3	71.1	57.7	4.85	0.023	n.s.	**0.00** **5**	0.046
	±13.1	±11.1	±7.3	±9.4	±14.6	**0.00** **1**				
1.1 Perspectives and attributes	60.2	65.1	67.8	66.9	50.8	4.45	n.s.	**0.00** **5**	**0.005**	**0.0** **10**
	±13.7	±10.7	±10.2	±10.2	±13.7	**0.0** **02**				
1.2. Relationships and roles	60.4	66.7	73.1	70.3	63.9	3.26	0.015	n.s.	n.s.	n.s.
	±11.5	±11.0	±7.5	±8.3	±13.7	0.015				
1.3 Emotional self-experience	58.8	64.8	72.4	67.9	50.6	6.28	0.020	**0.00** **8**	**0.00** **1**	**0.00** **6**
	±11.8	±12.2	±7.9	±13.0	±11.9	**0.001**				
Domain 2 - Incoherence	56.9	62.9	68.4	64.8	52.3	4.70	0.036	0.046	**0.004**	n.s.
	±9.4	±9.5	±9.5	±12.5	±14.6	**0.00** **2**				
2.1 Self-concepts	56.8	62.9	69.2	67.3	53.2	5.43	0.016	n.s.	**0.00** **3**	0.014
	±11.7	±9.4	±8.2	±11.3	±13.7	**0.00** **1**				
2.2 Autonomy	57.4	60.3	63.6	59.1	48.7	3.23	n.s.	0.024	0.011	n.s.
	±6.3	±10.0	±12.5	±14.0	±11.3	0.016				
2.3 Cognitive self-experience	52.9	60.3	66.7	63.1	54.3	3.81	**0.0** **10**	n.s.	n.s.	n.s.
	±10.2	±10.4	±8.6	±13.7	±15.2	**0.0** **06**				

**Table 5:** Pearson´s correlation coefficients between JTCI scales, AIDA total scale, internalizing scale of Youth Self Report (YSR), and BMI and body-related scales (EDI-2) in the examined groups. Only the JTCI and YSR scales including significant values are listed (α-level ≤ 0.01), ** *p* < 0.01 (bold print), *p* < 0.05 (light print), n.s. = not significant. Abbreviations: HA (harm avoidance), P (persistence), SD (self-directedness), ID (identity diffusion), and IP (internalizing problems).

	Groups	JTCI			AIDA	YSR	BMI
		HA	P	SD	ID	IP	kg/m^2^
**Drive for thinness**	AN_R_	n.s.	n.s.	−0.530	n.s.	n.s.	0.580
	DD	0.377 **	n.s.	**−0.5** 78 **	**0.4** 82 **	**0.** 428 **	n.s.
	BPD	n.s.	−0.599	n.s.	**0.6** 81 **	**0.** 680 **	n.s.
	ADHD	n.s.	n.s.	−0.683 **	n.s.	n.s.	n.s.
	CD	n.s.	n.s.	n.s.	n.s.	n.s.	n.s.
**Bulimia**	AN_R_	n.s.	n.s.	n.s.	0.518	n.s.	n.s.
	DD	**0.** 525 **	n.s.	**−0.5** 37 **	**0.** 493 **	**0.4** 45 **	0.366
	BPD	n.s.	−0.533	n.s.	0.703 **	0.658	n.s.
	ADHD	n.s.	n.s.	n.s.	0.574	n.s.	n.s.
	CD	n.s.	n.s.	n.s.	n.s.	n.s.	n.s.
**Body dissatisfaction**	AN_R_	0.499	n.s.	−0.701 **	n.s.	0.608	0.587
	DD	**0.5** 41 **	**−0.4** 74 **	**−0.6** 35 **	**0.5** 65 **	**0.5** 65 **	**0.4** 04 **
	BPD	n.s.	−0.563	n.s.	n.s.	n.s.	n.s.
	ADHD	n.s.	n.s.	−0.621	n.s.	n.s.	n.s.
	CD	n.s.	n.s.	n.s.	n.s.	n.s.	n.s.

**Table 6:** Hierarchical linear regression analysis with stepwise selection (identity diffusion = dependent variable) in mental-disorder groups. B = unstandardized beta, β = standardized coefficient.

Group	Model	*R*	Adjusted *R^2^*	*R^2^* Change	*df*	F	Sig. F Change	Coeffficients			
								B	β	t	*p*
AN_R_	Self-directedness	0.844	0.693	0.713	1	34.78	0.001	−0.733	−0.844	−5.897	0.001
DD	Self-directedness	0.761	0.570	0.580	1	57.96	0.001	−0.769	−0.761	−7.613	0.001
BPD	Bulimia	0.703	0.452	0.494	1	11.73	0.005	0.555	0.703	3.425	0.005
ADHD	Self-directedness	0.871	0.739	0.756	1	37.82	0.001	−0.754	−0.871	−6.150	0.001
CD	Harm avoidance	0.885	0.752	0.783	1	25.22	0.002	1.109	0.885	5.022	0.002

## Data Availability

The data presented in this study are available on reasonable request from the corresponding authors.
